# Lateral Femoral Cutaneous Nerve Angiomyoma

**DOI:** 10.7759/cureus.18726

**Published:** 2021-10-12

**Authors:** Luke Mugge, Danielle D Dang, Sidhartha Chandela

**Affiliations:** 1 Neurological Surgery, Inova Neuroscience and Spine Institute, Falls Church, USA; 2 Neurological Surgery, Inova Fairfax Medical Campus, Falls Church, USA; 3 Neurosurgery, Inova Neuroscience and Spine Institute, Falls Church, USA

**Keywords:** angiomyoma, angioleiomyoma, vascular leiomyoma, sensory nerve, smooth muscle, lateral femoral cutaneous nerve

## Abstract

Angiomyomas are benign tumors derived from smooth muscle cells of vessels. They are principally a pathology of the peripheral nervous system where they involve cutaneous nerves, causing pain and paresthesia. We present a case of a lateral femoral cutaneous nerve angiomyoma and its surgical treatment. A 24-year-old female presented to clinic with right thigh pain in the distribution of the lateral femoral cutaneous nerve, which had been ongoing and progressive for seven months. There was associated numbness and tingling. A lesion was noted in the anterior subcutaneous tissues of the thigh on contrasted CT and MRI. The patient was taken to the operating room where a pearly white lesion was found within the subcutaneous tissue. No effect was seen with stimulation of the lesion. The lesion was removed en bloc, and pathological analysis revealed an angiomyoma. Post-operatively, the patient reported complete resolution of all symptoms, namely, pain and paresthesia. No similar reports were identified within the literature. Together, angiomyomas have been described within the lower extremities to cause pain and paresthesia. This is the first reported case of an angiomyoma involvement within the lateral femoral cutaneous nerve. Complete surgical resection, in this case, was curative and diagnostic.

## Introduction

Angiomyoma is a smooth muscle neoplasm. It is thought to derive from the tunica media of small veins [1-2]. Other names include vascular leiomyoma or angioleiomyoma. These are classically benign lesions that develop within the subcutaneous tissue. From a diagnostic standpoint, these lesions present a challenge as they lack distinctive features on imaging, causing them to be one of many within a broad differential diagnosis [1]. On MRI, these lesions are reported to be both hyper and iso-intense to skeletal muscle [3], but is not altogether specific. The clinical presentation of an angiomyoma is fairly consistent, usually presenting as a solitary, painful mass within the subcutaneous tissues in an extremity.

In terms of incidence, 89% occur within the extremities [4]. Expansion to involve the median nerve [5-7], ulnar nerve [8-9], and radial nerve [10-11] have all been described. Angiomyomas similarly involve nerves of the lower extremities and have been reported in the sciatic nerve [12]. By and large, angiomyomas have a predilection for affecting females in the lower extremities [13], and in particular, involving the knee [14-15]. Involvement in the plantar aspect of the heel has also been described [16].

Herein, we present a case of an angiomyoma involving a branch of the lateral femoral cutaneous nerve. This is the first report of an angiomyoma involvement in this region of the body and with this nerve. In our case, the patient presented with uncontrolled pain and paresthesia. Here, complete surgical resection, in this case, was curative and diagnostic.

## Case presentation

Presentation

**Figure 1:**
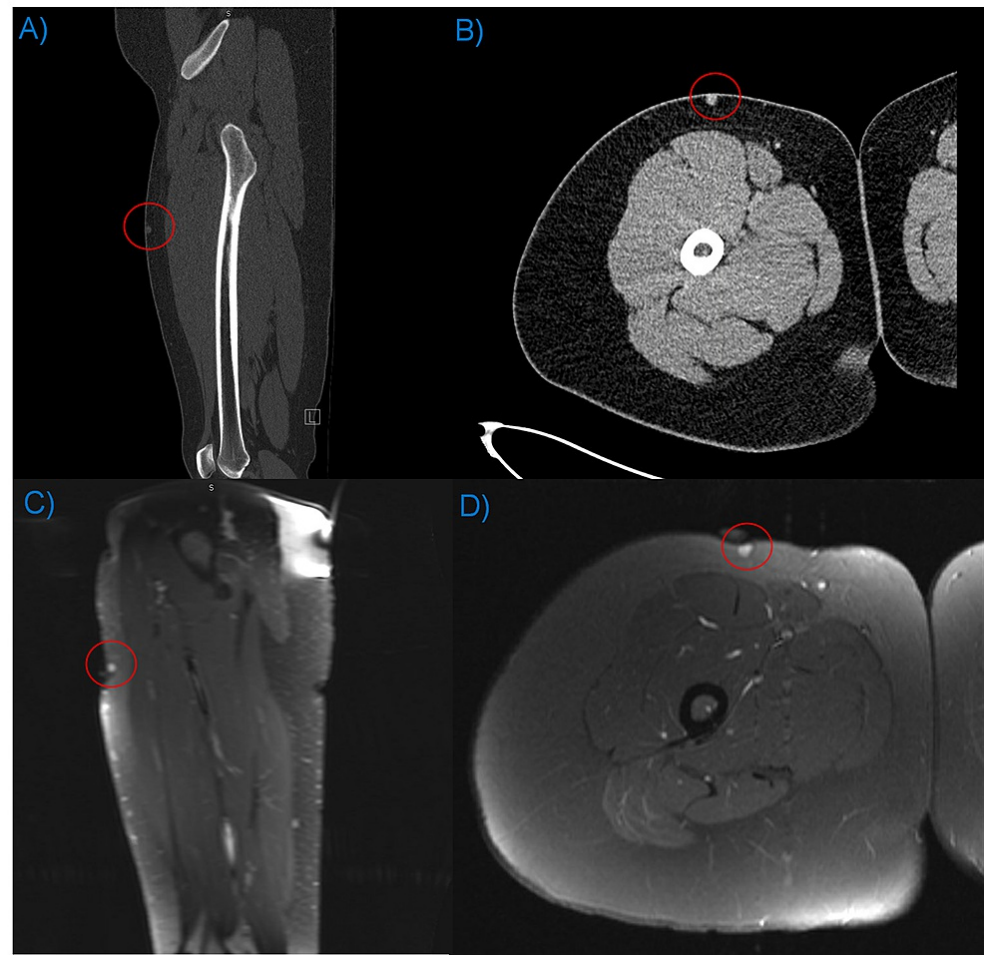
Pre-operative CT and MRI. Contrasted CT and MRI of the right lower extremity; A) sagittal contrasted CT, B) axial contrasted CT, C) sagittal T1 post-contrast MRI, D) axial T1 post-contrast MRI

**Figure 2:**
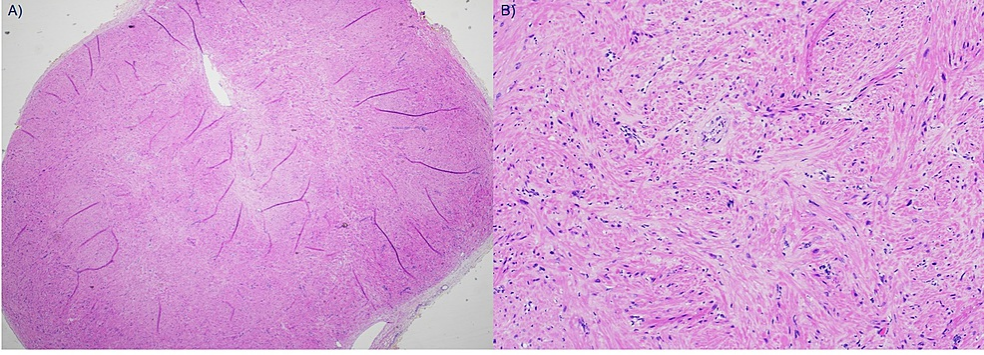
Histopathology. Representative images of the pathology of lesion: A) lower power, B) higher power

The patient is a 24-year-old female who presented with intractable and progressive pain in her right thigh for two weeks. She described this pain as shooting into her right leg with associated numbness and tingling. She also had subjective weakness in that same leg, causing her to walk with an antalgic gait. The patient reported feeling a slowing growing lump in her thigh for approximately seven months associated with intermittent pain. She was referred to the neurosurgery clinic for further evaluation. Workup included electromyography, which was unremarkable, and a contrasted CT and MRI (Figures 1-2, respectively). On presentation in the clinic, she indicated that the pain was so severe that she could no longer drive or take care of her family. Therapeutically, she was taking ibuprofen daily along with Vicodin. She was also taking gabapentin over the past week. All with no effect.

On exam, a palpable, hard, rubbery, lump was noted within the right anterior thigh. It was extremely tender to palpation with a positive Tinel’s sign. Neurologically, the patient did have paresthesia and tingling over the right anterior thigh and dorsum of the foot. No motor weakness was appreciated on objective testing. 

Given the clinical presentation, diagnostic testing, and image findings, the patient underwent surgical resection of the lesion.

Differential diagnosis

With this presentation, a number of pathologies were taken into consideration. Neoplasms to be considered were schwannoma, angiomyoma, or rhabdomyoma. Other etiologies included abscess, neuroma secondary to prior trauma, or seroma, along with others.

Radiology

Both CT and MRI were used for assessment of the lesion pre-operatively. Post-contrast CT revealed an enhancing lesion within the soft tissue of the anterior right thigh. Contrasted MRI redemonstrated an enhancing lesion within the same area. Here the lesion was homogeneously enhancing. Because of its size and peripheral location, a marker was used for localization. Representative images are presented in Figure 1.

Operative intervention

The patient was brought to the operating room and placed under general anesthesia. The patient was positioned supine. The right anterior thigh mass was palpated and incision was centered on the mass after which the area was prepared and draped in the usual fashion. The skin was incised with a 10 blade and dissection of the subcutaneous tissue was carried out using a combination of bovie cautery and blunt dissection using metzenbaum scissors. The mass was easily identified under the subcutaneous fat. This lesion was a solitary, pearly white mass located outside the fascia. The mass was circumferentially dissected and mobilized with afferent and efferent nerves were identified. An electromyography probe was used to stimulate the mass and both afferent and efferent nerves without effect, indicating that this had no motor component. Afterward, the afferent and efferent nerves were ligated and divided allowing for en bloc resection. The wound was irrigated and closed. Blood loss was minimal. The patient was taken to recovery and discharged later that day without incident or complication.

Pathology

Analysis was undertaken of the nodule which was 0.8 cm x 0.7 cm in size. Smooth muscle was seen throughout. The lesion was also highly vascularized. Together, these features supported a diagnosis of angiomyoma. Representative images are provided in Figure 2.

Post-operative care

The patient was seen in the clinic 10 days post-operatively and was noted to be doing well. She noted only incisional discomfort at that time. The patient reported complete resolution of symptoms at all subsequent visits.

## Discussion

Herein, we present a case of an anterior branch of the lateral femoral cutaneous nerve involvement with an angiomyoma. The patient presented with debilitating pain, with an exam notable for a positive Tinel’s sign. En bloc resection was successful and pathology rendered a diagnosis. Surgery was curative for our case. This is the only such case of an angiomyoma within this location and involving this nerve.

Pathologically, angiomyomas are known to derive from the smooth muscle found within the tunica media of veins as previously stated [1-2]. More specifically, it is a muscular coat that surrounds blood vessels with only sparse unmyelinated nerve fibers running with the arterioles [17]. A study by Hasegawa et al. demonstrated that nerve fibers can be seen within the tumor capsule and stroma on pathology when the main presenting symptom is pain [18]. The association of nerve involvement with a tumor capsule and pain for angiomyomas has been similarly confirmed in other studies [19]. More specifically, a study by Geddy et al. examined mast cell density in relation to incidents of painful angiomyomas determining that mast cell density was actually decreased in painful lesions, suggesting that mast cell degranulation may have occurred and potential local inflammation contributed to the development of lesion associated pain [20].

The common presentation of angiomyomas as a nodule, with or without associated pain, does not adequately distinguish this pathology from others in a differential diagnosis. Imaging, while useful in ruling out more malignant processes, is not without shortcomings in its capacity to describe and diagnose this pathology. The three main imaging modalities typically employed include ultrasonography, CT, and MRI, each providing less than ideal findings. A study by Ogata et al. exemplified the shortcomings of ultrasound’s diagnostic utility demonstrating that no specific differences between a schwannoma and angioleiomyoma were identifiable [21]. These lesions are detectable on contrasted CT as was the case with our study. Scott et al. reported a case where an angiomyoma was reported to be the cause of pain for an 11-year-old but where the scan needed to be extended further caudally than first anticipated [12]. MRI can also be useful diagnostically. Gupte et al. demonstrated that when a linear or branching pattern is seen on T2 weighted and short tau inversion recovery (STIR) sequencing, an angiomyoma should be included in the differential. [22]

As stated above, angiomyomas should be considered when soft tissues masses are observed within the upper extremities, and in particular the hands [23-24]. Interestingly, angiomyomas, when found to occur in the hand, are not commonly painful, which could give insight into the pathology and diagnosis [25]. Less common presentations of angiomyomas include those on the face or when there is intra-cranial involvement. Lesions involving the face are typically painless and surgery is curative [26]. When there is intra-cranial involvement, neurological dysfunction is usually the presenting symptom. Magliulo et al. described a case wherein a patient presented with a facial palsy secondary to involvement of an angiomyoma within the geniculate ganglion [27]. Finally, angiomyomas can occur in other intra-cranial locations where they affect nerve function as exemplified by Delgado-Fernandez et al. who described the involvement of an infratentorial lesion, presenting with hearing loss, but found to be amendable to surgical resection [28].

Together, surgical resection remains the mainstay for treatment for angiomyomas regardless of location or presentation. Within the literature and for our case, surgical resection successfully ameliorated symptoms with minimal need for medication post-operatively. Given the benign nature of this pathology and the success of surgery by treating it with relatively minimal risks and side effects, surgery should be recommended whenever possible for this pathology. This is particularly true in cases like this where sensory components were the only nerve fibers involved in the lesion, rendering resection safe without risking the development of iatrogenic neurological deficits.

While our case does describe a successful therapeutic intervention, our study is not without limitations or caveats. As a single case report, it provides insufficient evidence to suggest surgery is the only therapeutic option for this pathology. Given this patient had failed prior medical management, it is unlikely anything other than resection would have been beneficial. Further research is needed in order to describe this pathology further when it occurs in this location and to confirm if our approach represents the best therapeutic intervention.

## Conclusions

We present a case of an angiomyoma involvement in a branch of the lateral femoral cutaneous nerve. This is the first such report of involvement of this pathology within this nerve. The presentation of this pathology was classic, presenting with pain and a palpable nodule. Radiographically, this lesion was similar to that which has been previously reported. Pathology was essential for diagnosis. In this case, complete surgical resection was curative. While rare and benign, angiomyomas are a cause of pain within the lower extremity and should be considered in the differential diagnosis for nodules in the extremity. These are benign in nature and can be resected completely in a safe manner, with complete resolution of symptoms.
